# Comment on “Responders to Platelet-Rich Plasma in Osteoarthritis: A Technical Analysis”

**DOI:** 10.1155/2017/8620257

**Published:** 2017-12-28

**Authors:** J. Magalon, M. Velier, P. Francois, H. Graiet, J. Veran, F. Sabatier

**Affiliations:** ^1^Cell Therapy Laboratory, Hôpital de la Conception, AP-HM, INSERM CIC BT 1409, Marseille, France; ^2^Vascular Research Center of Marseille, Aix-Marseille University, INSERM UMR 1076, Marseille, France

We have read with great interest the recent review article of Milants et al. [1] published in BioMed Research International titled “Responders to Platelet-Rich Plasma in Osteoarthritis: A Technical Analysis.” We would like to congratulate the authors of this article, who have performed an extensive analysis of 19 randomized controlled trials assessing Platelet-Rich Plasma (PRP) in knee osteoarthritis and tried to point out characteristics of the procedure that could influence the clinical outcome. They have finally deeply studied 11 from the 19 studies corresponding to either bad responders (4 studies) or very good responders (7 studies) based on the level of the minimum clinically important improvement of the functional score used. Thus, 8 studies were not included in the technical analysis. The authors were able to conclude that a “platelet concentration lower than 5 times the baseline and avoidance of leukocytes should be preferred” for this specific indication. However, we think that using platelet and leukocyte concentrations or their increase ratio compared to blood as the standard to describe biological characteristics of PRP products is a “historical” mistake. This aspect was strengthened by the manufacturers of medical devices who have rapidly highlighted the increase factor in platelets compared to whole blood as the gold standard to describe PRP therapy and a race between devices to obtain the most concentrated PRP has begun. The introduction by Ehrenfest et al. [2, 3] of the notion of leukocyte-rich PRP and pure PRP based on the leukocyte concentration compared to whole blood baseline leukocyte level has given rise to PRP classification systems [4, 5], but none of these classifications have been widely adopted. The weakness related to the use of concentration is based on the fact that it does not take into account the final volume of the preparation and finally probably minimizes the difference between preparations. Indeed, data from our department showed that PRP prepared with the same technique can lead to PRP with similar platelets increase factor but with a 2- to 3-fold increase in platelet dose (Figure 1). Furthermore, platelet dose is simply obtained by multiplying the volume of PRP injected by its platelet concentration. The confusion linked to the use of platelet concentration instead of platelet dose has already furnished some interesting results in the literature. In 2011, Kaux et al. showed that Plateltex® device provided a highly concentrated PRP, namely, 3.5 times the whole blood baseline, as well as the lowest platelet dose (280 million on average) due to a very small amount of volume obtained (0.34 mL) [6]. The application of the “dose concept” to the technical analysis of Milants et al. gives also rise to surprising results. As initial blood counts of patients were not mentioned in any of the 11 selected studies in the analysis, we made the approximation that initial platelet count was 250 G/L. Thus, in the studies classifying PRP as ineffective, Filardo et al. have potentially used either 18.75 billion of platelets in their 2012 study [7] or 17.4 billion of platelets in their 2015 study [8], in a three-injection procedure. The total dose injected by Napolitano et al. [9] was around 9 billion and was also classified as ineffective. Conversely, the dose seems to be lower in the studies classified as effective; Patel et al. [10] have injected 2.4 or 4.8 (if two injections were performed) billion platelets, whereas Say et al. [11] have injected only 2.5 billion platelets. This “dose response” is supported by the fact that platelet dose in different PRP preparation is correlated with the main regenerative growth factors [12] and suggests that, in knee osteoarthritis, more is not necessarily better.

Also, the current description of PRP does not take into account PRP as a global product containing not only platelets and leukocytes but also red blood cells (RBCs). However, presence of RBCs was mentioned in only 1 of the 19 studies described and 0 of the 11 selected for the technical analysis. This was highlighted by the authors but we would like to emphasize this point because (i) deleterious clinical impact of RBCs on joints is clearly established with the model of hemophilic arthropathy [13] and (ii) the essential challenge of PRP preparation is to remove RBCs and reverse the initial composition of blood (95% of RBCs) and this is not achieved at all as some available devices furnish more RBCs than platelets in their PRP [14].

To conclude, we share with the authors the fact that, for each defined indication, a specific formulation of PRP should be required. However, we support recent findings from Chahla et al. [15] showing that “the current reporting of PRP preparation and composition does not enable comparison of the PRP products being delivered to patients. A detailed, precise, and stepwise description of the PRP preparation protocol is required to allow comparison among studies and provide reproducibility.”

Through this letter, the aim of the authors is to highlight two recurrent and major weaknesses in the majority of PRP-based regenerative therapy clinical reports or trials, which omit the dose-effect concept and minimize the impact of RBCs on joints. We strongly support the notion that the main learned societies working in the PRP field should work together to find an international consensus on the minimal PRP characterization required prior to injection, at least for level one studies, and encourage the use of systematic quality control and traceability data with an injection report for patients in daily use.

## Figures and Tables

**Figure 1:**
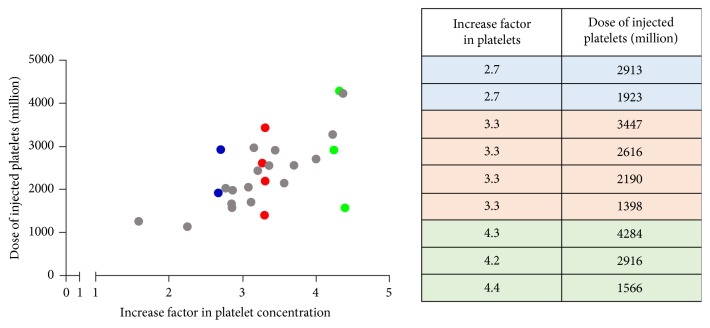
Graphic representation of different PRP preparations (*n* = 26) with increase factor in platelets (*x* axis) and dose of injected platelets (*y* axis). PRP prepared with the same technique can lead to preparations with similar increase factor (IF) in platelets (blue, red, and green points, resp., present IF of 2.7, 3.3, and 4.3), but displaying highly variable dose of injected platelets.
